# The Push-Pull Mentoring Model: Ensuring the Success of Mentors and Mentees

**DOI:** 10.2196/48037

**Published:** 2023-05-25

**Authors:** Julie K Silver, Nara Gavini

**Affiliations:** 1 Spaulding Rehabilitation Hospital Charlestown, MA United States; 2 Department of Physical Medicine and Rehabilitation Harvard Medical School Boston, MA United States; 3 Massachusetts General Hospital Boston, MA United States; 4 Brigham and Women's Hospital Boston, MA United States; 5 MGH Institute of Health Professions Boston, MA United States

**Keywords:** mentorship, mentor, medical education, diversity, equity, Push-Pull Mentoring Model

## Abstract

Mentorship is vital for professional development in academic research and clinical practice, yet it faces challenges due to a limited number of experienced mentors and a lack of protected time for mentorship that may disproportionately affect women mentors in midcareer who are doing much of this “invisible work.” The Push-Pull Mentoring Model offers a potential solution by emphasizing shared responsibility and active engagement between mentors and mentees; it fosters a flexible and collaborative approach that is mutually (though not necessarily equally) supportive of both individuals’ career goals, with mentees pushing mentors up and facilitating opportunities in their realm of influence, including but not limited to sponsorship, while mentors are simultaneously pulling them up. The Push-Pull Mentoring Model provides a promising alternative to traditional mentoring models and may help institutions address the challenges associated with limited mentorship resources.

## Introduction

In this paper, we describe a Push-Pull Mentoring Model (PPMM) that focuses on a “shared responsibility for the mentor-mentee relationship that is mutually, though not necessarily equally, supportive of both individuals’ career goals with mentees pushing mentors up and facilitating opportunities in their realm of influence, including but not limited to sponsorship, while mentors are simultaneously pulling them up” [1]. Bidirectional support for mentors and mentees takes into account that while being a mentor is largely considered a positive endeavor, there is a problem with supply and demand. Since there are not enough mentors and they often do this work without protected time or compensation, it is not surprising that mentoring others may be contributing to disparities in career advancement for women [2], especially if they identify with underrepresented groups (eg, race, ethnicity, disability, gender identity, and sexual orientation).

The PPMM is a novel approach that may allow mentors to receive additional career support from mentees who, as they ascend in their own careers, have increasing influence and power. This model utilizes *sponsorship*, which can be defined as “an action or a process in which someone, generally though not exclusively with more influence and power than the person they are sponsoring, facilitates an opportunity for an individual that might lead to career benefits including, but not limited to, accomplishments required for academic rank promotion and that can be documented on a curriculum vitae” [1]. This definition of *sponsorship* recognizes that mentees may have opportunities to sponsor mentors in various ways. For example, during training, someone might have an opportunity to suggest speakers, and then as the mentee becomes an attending physician, they may be the one selecting speakers. Mentees can also nominate mentors for recognition awards and invite them to be coauthors of publications now and in the future. Although the PPMM is not a standalone approach and should be incorporated into existing models, it provides a novel way for mentors to receive additional career support from mentees who have increasing influence and power. However, it does not solve all of the issues related to mentoring, such as providing mentors with protected time and compensation for their valuable work.

## Reimagining Mentorship

In a report titled *The Science of Effective Mentorship in STEMM* that was published by the National Academies of Sciences, Engineering, and Medicine [3], the experts noted that talent is equally distributed across sociocultural groups, but access and opportunity are not. The report defines *mentorship* as a “professional, working alliance in which individuals work together over time to support the personal and professional growth, development, and success of the relational partners through the provision of career and psychosocial support.” The conventional dyad of a strong and supportive mentor-mentee relationship that typically lasts for years, and sometimes even throughout someone’s career, can foster professional growth and advancement, as well as personal fulfillment, for both individuals. Although the report notes that this model continues to work well for some people, best practice mentoring programs offer multidimensional mentoring opportunities that incorporate, but are not limited to, the dyad model (Textbox 1). A systematic review, which included 31 studies that focused on underrepresented physicians and trainees in academic medicine, found that the dyad model was the most common but that novel models were also being successfully implemented [4].

**Enhancing mentorship programs.**

The Push-Pull Mentoring Model may be incorporated into the following strategies:

- *Develop comprehensive training programs:* Institutions should create workshops, training sessions, and mentorship-focused courses to equip mentees with the necessary skills and knowledge to become effective mentors. These programs should emphasize active listening, empathy, goal setting, and providing constructive feedback.
- *Encourage peer mentorship:* Institutions should promote peer mentorship among current mentees, providing opportunities for them to develop their mentoring skills and gain valuable experience in guiding others. This involvement not only contributes to their growth as future mentors but also fosters a culture of mentorship within the institution.
- *Establish mentor-mentee partnerships with clear expectations and goals:* Institutions should facilitate mentor-mentee partnerships that have well-defined expectations and goals. By working together to set specific objectives and track progress, mentees can gain insights into the qualities and skills needed to become effective mentors.
- *Provide opportunities for mentees to observe experienced mentors in action:* Institutions should create opportunities for mentees to shadow successful mentor-mentee relationships, observe meetings, and participate in mentorship-related events. By learning from real-life examples, mentees can better understand the practical aspects of being a mentor.
- *Ensure institutional support and recognition:* Institutions should provide adequate support structures, resources, and recognition for mentorship efforts. This can be achieved through financial incentives, mentor awards, or other forms of recognition that demonstrate the value of mentorship within the institution.

## Women and Mentorship

Mentoring plays a crucial role in the development of both trainees and faculty in academic medicine; however, women and people from underrepresented groups often lack mentoring opportunities in their own careers and, in some instances, may be carrying a disproportionately high responsibility for mentoring others. For example, a recent systematic review that included 91 studies found mentoring-related disparities for women in academic medicine that negatively affect their careers [5]. Organizations aiming to recruit women in surgery should recognize that a lack of mentoring or ineffective mentoring is a barrier [6,7]. Today’s academic medicine workforce is faced with numerous financial drivers that undermine models of unprotected and uncompensated mentoring. Indeed, many faculty members face unprecedented levels of personal education debt and must strive to achieve financial metrics at work that focus on grant funding and clinical productivity, which may affect their income, ability to pay their debt, and career progression.

The field of academic medicine has a scarcity of experienced and capable mentors across the research and clinical practice landscape, and this shortage can significantly impact the professional development and well-being of mentees. Regardless of gender, limited resources, inadequate support structures, and a lack of recognition for the value of mentorship may discourage experienced professionals from engaging in mentoring relationships. However, gender and racial disparities can further exacerbate the challenges faced in mentorship within academic research and clinical practice. Women and people who identify with underrepresented groups may face unique barriers to accessing quality mentorship, such as implicit (unconscious) bias, stereotype threat, and a lack of diverse mentors [8]. The yearslong COVID-19 pandemic has made access to mentors even more difficult, especially for women from underrepresented groups [9]. Limited mentor availability may contribute to feelings of isolation, stress, and burnout among mentees, hindering their personal and professional growth. Additionally, mentors who are overworked face an increased risk of burnout, negatively impacting their ability to provide effective guidance and support to their mentees. Institutional factors also contribute to the challenges faced by mentors and mentees in academic research and clinical practice. These disparities can hinder the professional development of individuals from underrepresented groups and perpetuate inequities.

Notably, women in midcareer often become marginalized and “invisible,” with their work and contributions being discounted or even dismissed altogether [10]. Women who are more senior in their careers face many of the same issues [11]. For example, women mentors who provide guidance on manuscripts and meet the criteria for coauthorship may have their scholarly contributions discounted as “helping,” with their names being listed in the *Acknowledgments* section rather than the byline or being unlisted in the paper altogether. Women might not be given credit for being mentors, even when they perform traditional mentorship activities, such as introducing mentees to key contacts within their professional networks, potentially opening doors for research collaborations, job opportunities, and career development. Especially for women and people from historically marginalized groups, these efforts may be considered “helping” rather than “real mentoring.” The repercussions are wide-ranging and may include marginalized mentors receiving lower ratings from trainees and not receiving recognition awards, as well as reduced compensation related to faculty-based metrics.

All relationships benefit from excellent communication, and both mentees and mentors should have ongoing conversations about their career goals and how best to support each other. As mentees become mentors themselves, these conversations will be invaluable.

## Conclusion

As the demand for effective mentorship in academic research and clinical practice continues to grow, it is essential to explore innovative approaches to addressing the challenges resulting from the limited number of experienced mentors. The PPMM offers a promising solution to this pressing issue, with the potential to improve outcomes for mentees, mentors, and institutions alike. The PPMM not only addresses the challenges associated with the limited number of experienced mentors but also provides a pathway for current mentees to become effective mentors themselves. By implementing these strategies, institutions can foster a culture of mentorship and ensure a continuous supply of skilled mentors for the future. The PPMM is a valuable addition to the existing models of mentoring and has the potential to transform the way mentorship is perceived and practiced in academic research and clinical practice.

## Abbreviations

PPMM: Push-Pull Mentoring Model
